# Optimal control dynamics of Gonorrhea in a structured population

**DOI:** 10.1016/j.heliyon.2023.e20531

**Published:** 2023-10-04

**Authors:** Joshua Kiddy K. Asamoah, Beilawu Safianu, Emmanuel Afrifa, Benjamin Obeng, Baba Seidu, Fredrick Asenso Wireko, Gui-Quan Sun

**Affiliations:** a School of Mathematics, North University of China, Taiyuan, Shanxi 030051, China; b Department of Mathematics, Kwame Nkrumah University of Science and Technology, Kumasi, Ghana; c Department of Mathematics, School of Mathematical Sciences, C. K. Tedam University of Technology and Applied Sciences, Navrongo, Ghana

**Keywords:** Optimal control analysis, Gonorrhea dynamics, Gonorrhea reproduction number, Structured population

## Abstract

Gonorrhea is a serious global health problem due to its high incidence, with approximately 82.4 million new cases in 2020. To evaluate the consequences of targeted dynamic control of gonorrhea infection transmission, a model for gonorrhea with optimal control analysis is proposed for a structured population. The study looked at the model's positively invariant and bounded regions. The gonorrhea secondary infection expression, $R_{0}$ for the structured population is computed. The maximum principle of Pontryagin is utilised to construct the optimal system for the formulated mathematical model. To reduce the continuous propagation of gonorrhea, we incorporated education, condoms usage, vaccinations, and treatment as control strategies. The numerical simulations show that the number of infections decreases when the controls are implemented. The effectiveness of the controls is shown using the efficacy plots.

## 1 Introduction

Gonorrhea is an infection that can be passed on through sexual contact. Gonorrhea can affect individuals of all genders. Gonorrhea commonly impacts the urethra, rectum, or throat. Gonorrhea has the potential to affect the cervix in females as well. Gonorrhea is typically transmitted through sexual activities involving the vagina, mouth, or anus [1], [2]. However, it is possible for infants born to infected mothers to acquire the infection during the birthing process. In infants, it is not uncommon for gonorrhoea to affect the eyes [1]. Engaging in abstinence, practising safe sex by using a condom, and being in a mutually monogamous relationship are widely recognised as effective measures for preventing transmitting infections through sex. Gonorrhea is brought on by the bacteria Neisseria gonorrhoea [1]. An estimated 106,000,000 new infections of gonorrhea among grownups worldwide, with a ten days incubation time for females and two to five days for males. If left untreated, a protracted gonorrhea infection can cause serious ocular infections, impotence in both sexes, premature births, unplanned abortions, stillbirths, and eventually death [1]. Between 1955 and 1965, the prevalence of gonorrhea was roughly level in some Western nations. The countries with a general increase in gonorrhea rates from 1965 to 1975 are the USA, Denmark, Canada, Norway, Finland, and the UK. Increased population mobility, more frequent partner changes, rising oral contraceptive use, declining condom and diaphragm use, and increasing gonococcal antibiotic resistance are all potential contributing factors to the rising rates [1]. Many gonorrhea infected people continue to be contagious until they receive antibiotic treatment because gonorrhea does not recover spontaneously after exposure for a long period [2]. Gonorrhea now affects more men than women, unlike chlamydia. Between 2010 and 2014, the gonorrhea infections in men climbed by 27.9%, whereas cases in women fell by 4.1% over that time. The increase in diagnoses of chlamydia and gonorrhoea among homosexual, bisexual, and other males who engage in sexual activity with men can be attributed to several factors [3]. Due to the continually high illness burden and the rising development of multidrug antibiotic resistance, infections caused by Neisseria gonorrhoeae (N. gonorrhoeae) have made gonorrhea to become a global health challenge. Developing new therapies is a crucial strategy for combating the threat posed by N. gonorrhoeae that is resistant to several common antibiotics [4]. Adamu et al. [5] conducted iteratively a theoretical investigation of gonorrhea patterns and gave some ideas that help understand the dynamics of gonorrhea. Most investigations on N. gonorrhoeae infection have used a straightforward mechanistic approach for the pathogen's spread in the population [6]. There exist several hypotheses that propose mechanisms for the spread of gonococcal infections. The models under consideration exhibit variations in their foundational presumptions about the fundamental components of transmission behaviour and the comparative fitness expenses associated with resistance and strain competition in a broader sense. Furthermore, these models also exhibit variations in their representation of sexual partnerships, encompassing compartmental, partnership, and individual-based models [7]. It is suitable to employ susceptible, infected, and recovered or the susceptible, exposed, infected, and recovered frameworks lacking vital phenomena (births and deaths) to mimic an epidemic of an infection in which healing confers lifelong antibodies. The work in [8] investigated a computational framework of gonorrhoea by segmenting the population into multiple groups to analyse the stability asymptotically. Additional data indicates that a substantial percentage of men are susceptible to contracting gonorrhoea when exposed to a considerable amount of the infection. This is supported by the observed increase in infection rates among males who engage in sexual activity with a single partner, with the incidence of infection growing in correlation with the number of exposures. [9]. Additionally, numerical simulation of a HIV and gonorrhea dynamic model revealed that a rise in gonorrhea infections in the presence of therapy leads to a drop in the compartment of gonorrhea and with an upward adjustment in the individuals who have only HIV [10]. Alcoholism is now a major health concern for society and a menace on a worldwide scale. A deterministic alcohol model with gonorrhea was developed, examined, and its fundamental characteristics were established by the work in [11]. A mathematical framework that is characterised by nonlinearity is used to examine the dynamics of syphilis spread in a heterogeneous environment with two stages of infection. The Lipschitz condition was employed by Oyeniyi et al. [12]. Using centre manifold theory and Lyapunov functions, the model's global stability and bifurcation analysis were performed, respectively. The authors of [13] performed a sensitivity analysis to determine how various factors–such as condom effectiveness, effective contact rate, condom compliance, progression rate, and treatment rate–affected their gonorrhea reproduction number, $R_{0}$. Adamu et al. [5] created a deterministic mathematical framework for the spread of N. gonorrhoeae infection and investigated how the only known control interventions-natural immunity and treatment-affected the disease's spread in a population. However, a conceptual model must take gonorrhea's specific epidemiologic characteristics into account. It is sufficient to consider only the community's sexually active members who might spread the disease to their connections. Optimal control model plays a significant role in the management of diseases. Several mathematical models have employed optimal control in a single population (see, [14], [15], [16], [17]). Therefore, this study builds on the work of [5] by developing a novel mathematical model with optimal control considering the heterogeneous (crisscrossed) population. Crisscrossed models are a slight extension of the generalized population model to account for the fact that gonorrhea can be transmitted through sexual activity between males and females, where the female infectives transmit the disease to a male susceptible and vice versa. And also with the fact that disease incubation may be different in males and females.

Following are the remaining parts of the paper: In Part 2, the mathematical model was developed. The mathematical deduction of the model without optimal controls is performed in Part 3. In Part 4, we presented the sensitivity analysis that led to the optimal control model described in Part 5. In Part 6, numerical simulations are presented. Part 7 contains the results and discussions. In Part 8, the study's conclusion is presented.

## 2 Model formulation

Based on the following works [18], [19], [20], we categorize the model into two population classes, males and females. Each class has four compartments: susceptibles, incubative, symptomatic and recovered. The number of susceptible females is constituted as $S_{f}(t)$, and the symptomatic female class is constituted as $I_{sf}(t)$, the incubative female class by $I_{cf}(t)$ and the treated recovered female class is denoted by $R_{f}^{T}(t)$. For the male population, susceptibles males are represented by $S_{m}(t)$, incubative males by $I_{cm}(t)$, the symptomatic male class by $I_{sm}(t)$ and the treated recovered male class is denoted by $R_{m}^{T}(t)$. The recruitment rate for females denoted by $\theta_{f}$ and that of the males by $\theta_{m}$, thus, we assumed that the female and male compartments have been populated only by the new females and males who enter the community, respectively. So that our model stays as structured throughout the simulation time. Similarly, the recovery rate in females is denoted as $\alpha_{f}$ whilst that of the males is represented by $\alpha_{m}$. The infectivity probability rate of symptomatic men and vulnerable women is $\eta_{sm}$, and the infectivity probability rate of incubative men and vulnerable women is $\eta_{cm}$. In the same vain, the infectivity rate of symptomatic females and vulnerable men is denoted as $\eta_{sf}$ and the infectivity rate of incubative female and susceptible males is denoted as $\eta_{cf}$. The new drive of transmission between males and females is denoted as $\beta_{mf}$, and that of females and males is denoted as $\beta_{fm}$. The net force of infection is denoted as $\lambda_{mf}$ and $\lambda_{fm}$, respectively. The proportion at which recovered females move to the incubative female class is given by $\left(1-\rho_{f}\right)$ and the rate at which females in the recovery relapse is given by $\kappa_{f}$. The proportion at which recovered males return to the incubative male susceptible class is $\left(1-\rho_{m}\right)$. The relapse rate at which males in the recovery class return to the incubative class is $\kappa_{m}$. Natural mortality rates are expressed as $\mu_{f}$ for females and $\mu_{m}$ for men. The rate at which males in the incubative class move to the incubative male class is given by $\gamma_{m}$ and that of the females by $\gamma_{f}$. The gonorrhea mortality rate in the female population is denoted as $\zeta_{f}$. That of the gonorrhea mortality rate in the male population is denoted as $\zeta_{m}$. The entire female class is calculated as: $N_{f}(t)=S_{f}(t)+I_{cf}(t)+I_{sf}(t)+R_{f}(t)$, and the entire male class is calculated as: $N_{m}(t)=S_{m}(t)+I_{cm}(t)+I_{sm}(t)+R_{m}(t)$. The provided model description outlines a set of nonlinear differential equations that serve as a predictable framework for characterising the pattern of transmission of gonorrhea within a population in equation (1).

(1) $$\left\{ \begin{array}{cc} \frac{dS_{f}}{dt} & =\theta_{f}-\lambda_{mf}S_{f}+\rho_{f}R_{f}^{T}-\mu_{f}S_{f},\\ \frac{dI_{cf}}{dt} & =\lambda_{mf}S_{f}+(1-\rho_{f})\kappa_{f}R_{f}^{T}-(\gamma_{f}+\mu_{f})I_{cf},\\ \frac{dI_{sf}}{dt} & =\gamma_{f}I_{cf}-(\alpha_{f}+\mu_{f}+\zeta_{f})I_{sf},\\ \frac{dR_{f}^{T}}{dt} & =\alpha_{f}I_{sf}-(\rho_{f}+(1-\rho_{f})\kappa_{f}+\mu_{f})R_{f}^{T},\\ \frac{dS_{m}}{dt} & =\theta_{m}-\lambda_{fm}S_{m}+\rho_{m}R_{m}^{T}-\mu_{m}S_{m},\\ \frac{dI_{cm}}{dt} & =\lambda_{fm}S_{m}+(1-\rho_{m})\kappa_{m}R_{m}^{T}-(\gamma_{m}+\mu_{m})I_{cm},\\ \frac{dI_{sm}}{dt} & =\gamma_{m}I_{cm}-(\alpha_{m}+\mu_{m}+\zeta_{m})I_{sm},\\ \frac{dR_{m}^{T}}{dt} & =\alpha_{m}I_{sm}-((1-\rho_{m})\kappa_{m}+\rho_{m}+\mu_{m})R_{m}^{T}, \end{array}\right.$$

where, $\lambda_{mf}=\frac{\beta_{mf}(\eta_{sm}I_{sm}+\eta_{cm}I_{cm})}{N_{f}}$, $\lambda_{fm}=\frac{\beta_{fm}(\eta_{sf}I_{sf}+\eta_{cf}I_{cf})}{N_{m}}$, and $S_{f}(t)>0,\;I_{cf}(t)\geq 0,\;I_{sf}(t)\geq 0,\;R_{f}^{T}(t)\geq 0,\;S_{m}(t)>0,\;I_{cm}(t)\geq 0,\;I_{sm}(t)\geq 0,\;R_{m}^{T}(t)\geq 0$.

## 3 Model analysis

### 3.1 Positivity and boundedness

Lemma 1 *The solution set*$\left(S_{f},I_{cf},I_{sf},R_{f}^{T},S_{m},I_{cm},I_{sm},R_{m}^{T}\right)$*is positively invariant and bounded in* Ω *=* $\left\{(S_{f},I_{cf},I_{sf},R_{f}^{T},S_{m},I_{cm},I_{sm},R_{m}^{T})\in R_{+}^{8}|N_{f}\leq \frac{\theta_{f}}{\mu_{f}}\;\;and\;\;N_{m}\leq \frac{\theta_{m}}{\mu_{m}}\right\}$*.*

Proof Let $\tau (y)=\{y(t)=0\;\;\text{and}\;\;(S_{f},I_{cf},I_{sf},R_{f}^{T},S_{m},I_{cm},I_{sm},R_{m}^{T})\in R^{8}\geq 0\}$, then $\forall y\in (S_{f},I_{cf},I_{sf},R_{f}^{T},S_{m},I_{cm},I_{sm},R_{m}^{T})$, we have;

$${\frac{dS_{f}}{dt}|}_{\tau (S_{f}=0)}=\theta_{f}+\rho_{f}R_{f}^{T}>0,\;\;\;{\frac{dI_{cf}}{dt}|}_{\tau (I_{cf}=0)}=\lambda_{mf}S_{f}+(1-\rho_{f})\kappa_{f}R_{f}^{T}\geq 0,\;\;\;{\frac{dI_{s_{f}}}{dt}|}_{\tau (I_{s_{f}}=0)}=\gamma_{f}I_{cf}\geq 0,{\frac{d_{R_{f}^{T}}}{dt}|}_{\tau (R_{f}^{T}=0)}=\alpha_{f}I_{s_{f}}\geq 0,\;\;{\frac{dS_{m}}{dt}|}_{\tau ({}_{S_{m}=0})}=\theta_{m}+\rho_{m}R_{m}^{T}>0,\;\;\;{\frac{dI_{cm}}{dt}|}_{\tau (I_{cm=0})}=\lambda_{fm}S_{m}+(1-\rho_{m})\kappa_{m}R_{m}^{T}\geq 0,{\frac{dI_{s_{m}}}{dt}|}_{\tau (I_{s_{m}=0})}=\gamma_{m}i_{cm}\geq 0,\;\;\;{\frac{dR_{m}^{T}}{dt}|}_{\tau (R_{m}^{T}=0)}=\alpha_{f}I_{s_{m}=0}\geq 0.$$

It can be seen that for all t ≥0, the solution set $S_{f},I_{cf},I_{sf},R_{f}^{T},S_{m},I_{cm},I_{sm},R_{m}^{T}$ will always be nonnegative, which indicates that, the system is positively invariant. Now, for the boundedness, considering the total female population, we have the following

$$\frac{dN_{f}}{dt}=\theta_{f}-\mu_{f}(S_{f}+I_{cf}+I_{sf}+R_{f}^{T})-\zeta_{f}I_{sf},\leq \theta_{f}-\mu_{f}N_{f},N_{f}(t)\leq \frac{\theta_{f}}{\mu_{f}}+e^{-\mu_{f}t}\left({N_{f}}_{0}-\frac{\theta_{f}}{\mu_{f}}\right),$$

as *t* approaches infinity, the exponential function also approaches zero. Therefore, if $0\leq N_{f}(0)\leq \frac{\theta_{f}}{\mu_{f}}$, then

$$0\leq \underset{t\to 0}{\lim\;\sup}\;N_{f}(t)\leq \frac{\theta_{f}}{\mu_{f}}.$$

Using a similar approach for the total male population, we have

$$0\leq \underset{t\to 0}{\lim\;\sup}\;N_{m}(t)\leq \frac{\theta_{m}}{\mu_{m}}.$$

The proof of Lemma 1 is now concluded, and this indicates that all the solution starting in Ω remains in Ω. Thus, leading to the boundedness of the model. Hence, the model is reasonable for an epidemiological study [21]. □

### 3.2 Scaling

In this section we employ scaling method to reduce our system complexity, scaling the above equation (1), with the transformations, $s_{f}=\frac{S_{f}}{N_{f}};\;i_{cf}=\frac{I_{cf}}{N_{f}};\;i_{sf}=\frac{I_{sf}}{N_{f}};\;r_{f}=\frac{R_{f}^{T}}{N_{f}}$ gives:

(2) $$\frac{ds_{f}}{dt}=\theta_{f}-\lambda_{mf}s_{f}+\rho_{f}r_{f}-\mu_{f}s_{f},\frac{di_{cf}}{dt}=\lambda_{mf}s_{f}+(1-\rho_{f})\kappa_{f}r_{f}-(\gamma_{f}+\mu_{f})i_{cf},\frac{di_{sf}}{dt}=\gamma_{f}i_{cf}-(\alpha_{f}+\mu_{f}+\zeta_{f})i_{sf},\frac{dr_{f}}{dt}=\alpha_{f}i_{sf}-(\rho_{f}+(1-\rho_{f})\kappa_{f}+\mu_{f})r_{f},\frac{ds_{m}}{dt}=\theta_{m}-\lambda_{fm}s_{m}+\rho_{m}r_{m}-\mu_{m}s_{m},\frac{di_{cm}}{dt}=\lambda_{fm}s_{m}+(1-\rho_{m})\kappa_{m}r_{m}-(\gamma_{m}+\mu_{m})i_{cm},\frac{di_{sm}}{dt}=\gamma_{m}i_{cm}-(\alpha_{m}+\mu_{m}+\zeta_{m})i_{sm},\frac{dr_{m}}{dt}=\alpha_{m}i_{sm}-(\rho_{m}+(1-\rho_{m})\kappa_{m}+\mu_{m})r_{m}.$$

After scaling the force of infection terms becomes, $\lambda_{mf}=\beta_{mf}(\eta_{sm}i_{sm}+\eta_{cm}i_{cm})$ and $\lambda_{fm}=\beta_{fm}(\eta_{sf}i_{sf}+\eta_{cf}i_{cf})$. With the same solution set;

$$\left\{(s_{f},i_{cf},i_{sf},r_{f},s_{m},i_{cm},i_{sm},r_{m})|s_{f}+i_{cf}+i_{sf}+r_{f}\leq \frac{\theta_{f}}{\mu_{f}};\;s_{m}+i_{cm}+i_{sm}+r_{m}\leq \frac{\theta_{m}}{\mu_{m}}\right\}.$$

### 3.3 Gonorrhea-free equilibrium point

The steady-states without infection may be investigated by turning the right-hand end of the model equations in the scaled system (2) to zero. It implies,

$$k=\{{s_{f}}^{⁎},{i_{cf}}^{⁎},{i_{sf}}^{⁎},{r_{f}}^{⁎},{s_{m}}^{⁎},{i_{cm}}^{⁎},{i_{sm}}^{⁎},{r_{m}}^{⁎}\}=\left\{\frac{\theta_{f}}{\mu_{f}},0,0,0,\frac{\theta_{m}}{\mu_{m}},0,0,0\right\}.$$

### 3.4 Gonorrhea reproduction number, $R_{0}$

The average number of individuals that can develop a disease spread via an infected individual in a community that is highly vulnerable or prone to being affected, is known as the gonorrhea reproduction number, or $R_{0}$. The frequency of contact with the host population, the possibility that a disease will spread through contact, and the infection length all impact the reproduction rate. Employing the next-generation matrix method, we construct an equation for $R_{0}$. The generation matrix's spectral radius (*ρ*), which has the nonnegative eigenvalue, is the gonorrhea replication (reproduction) number, $R_{0}$. i.e. $\rho (FV^{-1})$, where *F* is the new infections and *V* constitute the transitional phrases. We consider only the disease classes;

(3) $$\frac{di_{cf}}{dt}=\beta_{mf}(\eta_{sm}i_{sm}+\eta_{cm}i_{cm})s_{f}+\rho_{f}r_{f}-(\gamma_{f}+\mu_{f})i_{cf},\frac{di_{sf}}{dt}=\gamma_{f}i_{cf}-(\alpha_{f}+\mu_{f}+\zeta_{f})i_{sf},\frac{di_{cm}}{dt}=\beta_{fm}(\eta_{sf}i_{sf}+\eta_{cf}i_{cf})s_{m}+\rho_{m}r_{m}-(\gamma_{m}+\mu_{m})i_{cm},\frac{di_{sm}}{dt}=\gamma_{m}i_{cm}-(\alpha_{m}+\mu_{m}+\zeta_{m})i_{sm}.$$

From the above equation (3) we have the following;

$$Ϝ=\left[\begin{array}{c} \beta_{mf}(\eta_{sm}i_{sm}+\eta_{cm}i_{cm})s_{f}\\ 0\\ \beta_{fm}(\eta_{sf}i_{sf}+\eta_{cf}i_{cf})s_{m}\\ 0 \end{array}\right],F=\left[\begin{array}{cccc} \frac{\partial f_{1}}{\partial i_{cf}} & \frac{\partial f_{1}}{\partial i_{sf}} & \frac{\partial f_{1}}{\partial i_{cm}} & \frac{\partial f_{1}}{\partial i_{sm}}\\ \frac{\partial f_{2}}{\partial i_{cf}} & \frac{\partial f_{2}}{\partial i_{sf}} & \frac{\partial f_{2}}{\partial i_{cm}} & \frac{\partial f_{2}}{\partial i_{sm}}\\ \frac{\partial f_{3}}{\partial i_{cf}} & \frac{\partial f_{3}}{\partial i_{sf}} & \frac{\partial f_{3}}{\partial i_{cm}} & \frac{\partial f_{3}}{\partial i_{sm}}\\ \frac{\partial f_{4}}{\partial i_{cf}} & \frac{\partial f_{4}}{\partial i_{sf}} & \frac{\partial f_{4}}{\partial i_{cm}} & \frac{\partial f_{4}}{\partial i_{sm}} \end{array}\right],$$

$$F=\left[\begin{array}{cccc} 0 & 0 & \beta_{mf}\eta_{cm}s_{f} & \beta_{mf}\eta_{sm}s_{f}\\ 0 & 0 & 0 & 0\\ \beta_{fm}\eta_{cf}s_{m} & \beta_{fm}\eta_{sf}s_{m} & 0 & 0\\ 0 & 0 & 0 & 0 \end{array}\right],$$

$$F(k)=\left[\begin{array}{cccc} 0 & 0 & \beta_{mf}\eta_{cm}\left(\frac{\theta_{f}}{\mu_{f}}\right) & \beta_{mf}\eta_{sm}\left(\frac{\theta_{f}}{\mu_{f}}\right)\\ 0 & 0 & 0 & 0\\ \beta_{fm}\eta_{cf}\left(\frac{\theta_{m}}{\mu_{m}}\right) & \beta_{fm}\eta_{sf}\left(\frac{\theta_{m}}{\mu_{m}}\right) & 0 & 0\\ 0 & 0 & 0 & 0 \end{array}\right].$$

Let $\delta_{1}=\beta_{mf}\eta_{cm}\left(\frac{\theta_{f}}{\mu_{f}}\right)$, $\delta_{2}=\beta_{mf}\eta_{sm}\left(\frac{\theta_{f}}{\mu_{f}}\right)$, $\delta_{3}=\beta_{fm}\eta_{cf}\left(\frac{\theta_{m}}{\mu_{m}}\right)$, $\delta_{4}=\beta_{fm}\eta_{sf}\left(\frac{\theta_{m}}{\mu_{m}}\right)$. Which implies

$$F(k)=\left[\begin{array}{cccc} 0 & 0 & \delta_{1} & \delta_{2}\\ 0 & 0 & 0 & 0\\ \delta_{3} & \delta_{4} & 0 & 0\\ 0 & 0 & 0 & 0 \end{array}\right].$$

Now, the transitional phrases matrix, *V* is obtained as:

$$\upsilon =\left[\begin{array}{c} -(1-\rho_{f})\kappa_{f}r_{f}+(\gamma_{f}+\mu_{f})i_{cf}\\ -\gamma_{f}i_{cf}+(\alpha_{f}+\mu_{f}+\zeta_{f})i_{sf}\\ -(1-\rho_{m})\kappa_{m}r_{m}+(\gamma_{m}+\mu_{m})i_{cm}\\ -\gamma_{m}i_{cm}+(\alpha_{m}+\mu_{m}+\zeta_{m})i_{sm} \end{array}\right],V=\left[\begin{array}{cccc} \frac{\partial v_{1}}{\partial i_{cf}} & \frac{\partial v_{1}}{\partial i_{sf}} & \frac{\partial v_{1}}{\partial i_{cm}} & \frac{\partial v_{1}}{\partial i_{sm}}\\ \frac{\partial v_{2}}{\partial i_{cf}} & \frac{\partial v_{2}}{\partial i_{sf}} & \frac{\partial v_{2}}{\partial i_{cm}} & \frac{\partial v_{2}}{\partial i_{sm}}\\ \frac{\partial v_{3}}{\partial i_{cf}} & \frac{\partial v_{3}}{\partial i_{sf}} & \frac{\partial v_{3}}{\partial i_{cm}} & \frac{\partial v_{3}}{\partial i_{sm}}\\ \frac{\partial v_{4}}{\partial i_{cf}} & \frac{\partial v_{4}}{\partial i_{sf}} & \frac{\partial v_{4}}{\partial i_{cm}} & \frac{\partial v_{4}}{\partial i_{sm}} \end{array}\right].$$

$$V=\left[\begin{array}{cccc} \left(\gamma_{f}+\mu_{f}\right) & 0 & 0 & 0\\ -\gamma_{f} & \left(\alpha_{f}+\mu_{f}+\zeta_{f}\right) & 0 & 0\\ 0 & 0 & \left(\gamma_{m}+\mu_{m}\right) & 0\\ 0 & 0 & -\gamma_{m} & \left(\alpha_{m}+\mu_{m}+\zeta_{m}\right) \end{array}\right].$$

Let $\sigma_{1}=(\gamma_{f}+\mu_{f})$, $\sigma_{2}=(\alpha_{f}+\mu_{f}+\zeta_{f})$, $\sigma_{3}=(\gamma_{m}+\mu_{m})$, and $\sigma_{4}=(\alpha_{m}+\mu_{m}+\zeta_{m})$.

$$V^{-1}=\left[\begin{array}{cccc} \frac{1}{\sigma_{1}} & 0 & 0 & 0\\ \frac{\gamma_{f}}{\sigma_{1}\sigma_{2}} & \frac{1}{\sigma_{2}} & 0 & 0\\ 0 & 0 & \frac{1}{\sigma_{3}} & 0\\ 0 & 0 & \frac{\gamma_{m}}{\sigma_{3}\sigma_{4}} & \frac{1}{\sigma_{4}} \end{array}\right].$$

$$FV^{-1}=\left[\begin{array}{cccc} 0 & 0 & \delta_{1} & \delta_{2}\\ 0 & 0 & 0 & 0\\ \delta_{3} & \delta_{4} & 0 & 0\\ 0 & 0 & 0 & 0 \end{array}\right]\times \left[\begin{array}{cccc} \frac{1}{\sigma_{1}} & 0 & 0 & 0\\ \frac{\gamma_{f}}{\sigma_{1}\sigma_{2}} & \frac{1}{\sigma_{2}} & 0 & 0\\ 0 & 0 & \frac{1}{\sigma_{3}} & 0\\ 0 & 0 & \frac{\gamma_{m}}{\sigma_{3}\sigma_{4}} & \frac{1}{\sigma_{4}} \end{array}\right]=\left[\begin{array}{cccc} 0 & 0 & \left(\frac{\delta_{1}}{\sigma_{3}}+\frac{\delta_{2}\gamma_{m}}{\sigma_{3}\sigma_{4}}\right) & \frac{\delta_{2}}{\sigma_{4}}\\ 0 & 0 & 0 & 0\\ \left(\frac{\delta_{3}}{\sigma_{1}}+\frac{\delta_{4}\gamma_{f}}{\sigma_{1}\sigma_{2}}\right) & \frac{\delta_{4}}{\sigma_{2}} & 0 & 0\\ 0 & 0 & 0 & 0 \end{array}\right].$$

$$|FV^{-1}-\lambda I|=\left|\begin{array}{cccc} -\lambda & 0 & \left(\frac{\delta_{1}}{\sigma_{3}}+\frac{\delta_{2}\gamma_{m}}{\sigma_{3}\sigma_{4}}\right) & \frac{\delta_{2}}{\sigma_{4}}\\ 0 & -\lambda & 0 & 0\\ \left(\frac{\delta_{3}}{\sigma_{1}}+\frac{\delta_{4}\gamma_{f}}{\sigma_{1}\sigma_{2}}\right) & \frac{\delta_{4}}{\sigma_{2}} & -\lambda & 0\\ 0 & 0 & 0 & -\lambda \end{array}\right|=0,$$

$$-\lambda [-\lambda (\lambda^{2})]+\left(\frac{\delta_{1}}{\sigma_{3}}+\frac{\delta_{2}\gamma_{m}}{\sigma_{3}\sigma_{4}}\right)\left[\lambda \left(-\lambda \left(\frac{\delta_{3}}{\sigma_{1}}+\frac{\delta_{4}\gamma_{f}}{\sigma_{1}\sigma_{2}}\right)\right)\right]=0,\lambda^{2}\left(\lambda^{2}-\left(\frac{\delta_{1}}{\sigma_{3}}+\frac{\delta_{2}\gamma_{m}}{\sigma_{3}\sigma_{4}}\right)\left(\frac{\delta_{3}}{\sigma_{1}}+\frac{\delta_{4}\gamma_{f}}{\sigma_{1}\sigma_{2}}\right)\right)=0,\lambda_{1,2}=0\;\;\text{or}\;\;\lambda^{2}-\left(\frac{\delta_{1}}{\sigma_{3}}+\frac{\delta_{2}\gamma_{m}}{\sigma_{3}\sigma_{4}}\right)\left(\frac{\delta_{3}}{\sigma_{1}}+\frac{\delta_{4}\gamma_{f}}{\sigma_{1}\sigma_{2}}\right)=0,\lambda^{2}=\left(\frac{\delta_{1}}{\sigma_{3}}+\frac{\delta_{2}\gamma_{m}}{\sigma_{3}\sigma_{4}}\right)\left(\frac{\delta_{3}}{\sigma_{1}}+\frac{\delta_{4}\gamma_{f}}{\sigma_{1}\sigma_{2}}\right).$$

Let

$$\xi_{1}=\left(\frac{\delta_{1}}{\sigma_{3}}+\frac{\delta_{2}\gamma_{m}}{\sigma_{3}\sigma_{4}}\right)=\left(\frac{\delta_{1}\sigma_{4}+\delta_{2}\gamma_{m}}{\sigma_{3}\sigma_{4}}\right),\text{and}\;\;\xi_{2}=\left(\frac{\delta_{3}}{\sigma_{1}}+\frac{\delta_{4}\gamma_{f}}{\sigma_{1}\sigma_{2}}\right)=\left(\frac{\delta_{3}\sigma_{2}+\delta_{4}\gamma_{f}}{\sigma_{1}\sigma_{2}}\right).$$

Therefore,

$$\lambda^{2}=\xi_{1}\xi_{2},\lambda_{3}=-\sqrt{\xi_{1}\xi_{2}},\lambda_{4}=\sqrt{\xi_{1}\xi_{2}}.$$

Since $\lambda_{4}>\lambda_{3}$, the gonorrhea reproduction number is given as

(4) $$R_{0}=\sqrt{\frac{\delta_{1}(\delta_{3}\sigma_{2}\sigma_{4}+\delta_{4}\sigma_{4}\gamma_{f})+\delta_{2}(\delta_{3}\sigma_{2}\gamma_{m}+\delta_{4}\gamma_{f}\gamma_{m})}{\sigma_{1}\sigma_{2}\sigma_{3}\sigma_{4}}}.$$

Equation (4) can be written explicitly as

(5) $$R_{0}=\sqrt{\frac{\beta_{fm}\;\beta_{mf}\;\theta_{f}\;\theta_{m}\;\left(\alpha_{f}\;\eta_{cf}+\eta_{sf}\;\gamma_{f}+\eta_{cf}\;\mu_{f}+\eta_{cf}\;\zeta_{f}\right)\;\left(\alpha_{m}\;\eta_{cm}+\eta_{sm}\;\gamma_{m}+\eta_{cm}\;\mu_{m}+\eta_{cm}\;\zeta_{m}\right)}{\mu_{f}\;\mu_{m}\;\left(\gamma_{f}+\mu_{f}\right)\;\left(\gamma_{m}+\mu_{m}\right)\;\left(\alpha_{f}+\mu_{f}+\zeta_{f}\right)\;\left(\alpha_{m}+\mu_{m}+\zeta_{m}\right)}}.$$

**Figure 1:**
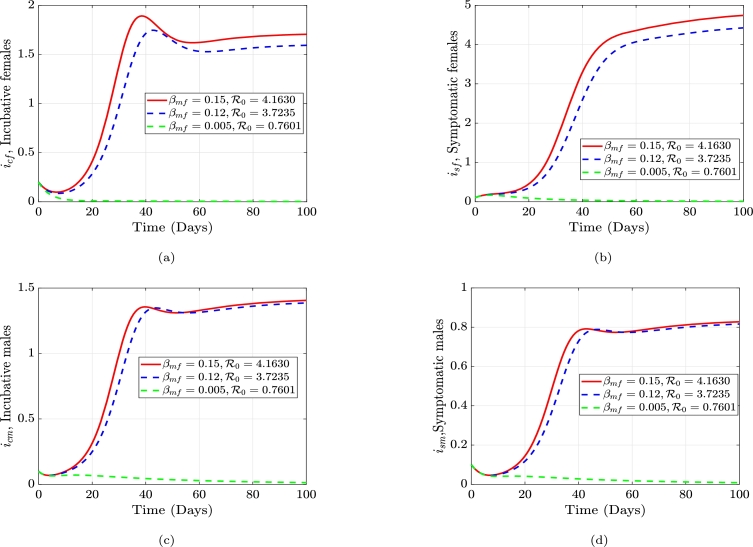
The effect of altering the male-to-female transmission rate, *β*_*mf*_, on the gonorrhea reproduction number and the infected females and males compartments, respectively.

**Figure 2:**
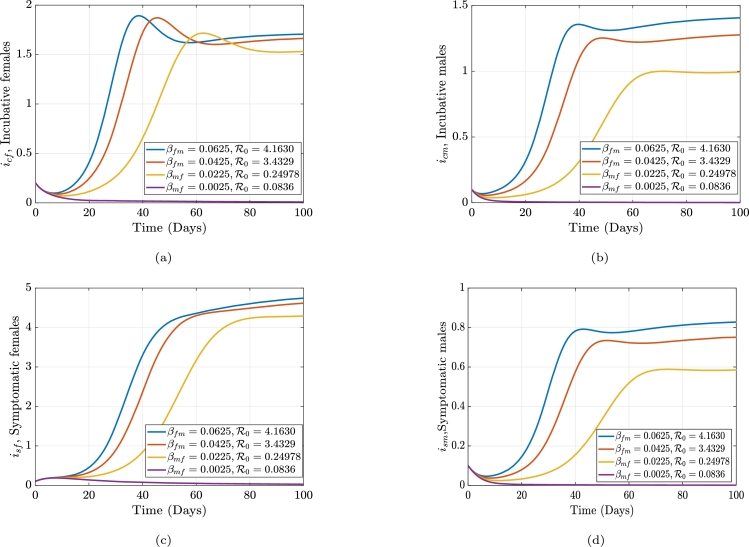
The effect of altering the female-to-male transmission rate, *β*_*fm*_, on the gonorrhea reproduction number and the infected females and males compartments, respectively.

**Figure 3:**
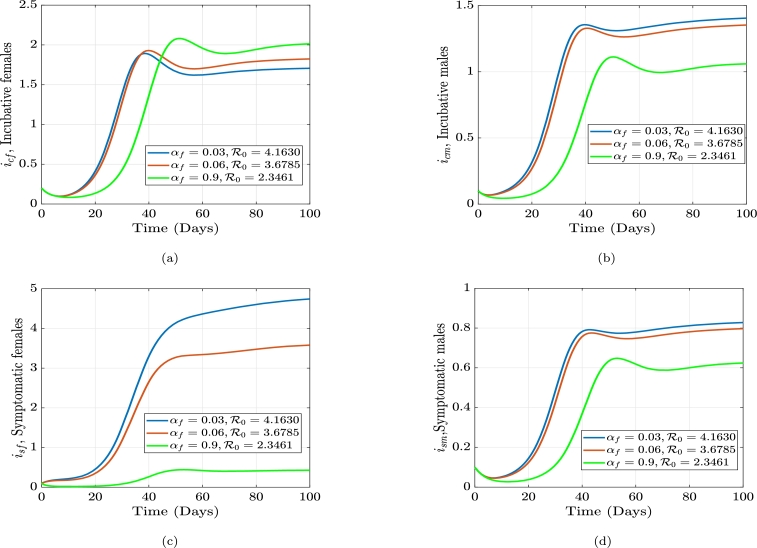
The effect of altering the female treatment rate, *α*_*f*_, on the gonorrhea reproduction number and the infected females and males compartments, respectively.

**Figure 4:**
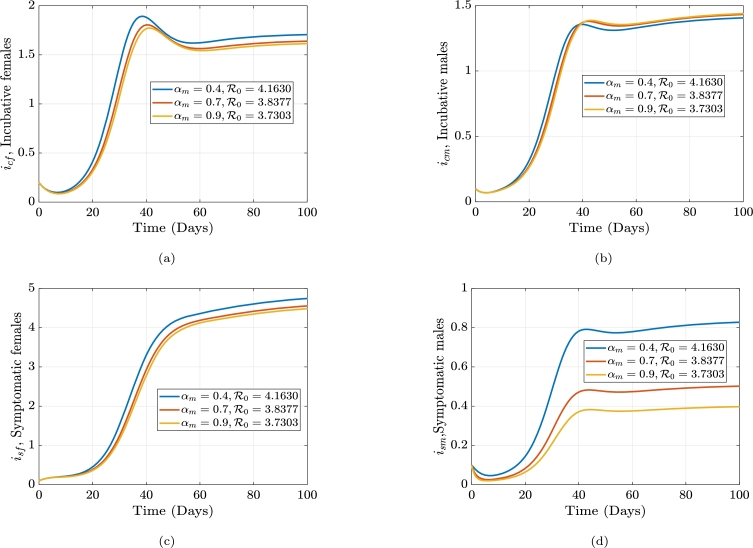
The effect of altering the male treatment rate, *α*_*m*_, on the gonorrhea reproduction number and the infected females and males compartments, respectively.

From equation (5), if $R_{0}<1$, the disease will die out in the population, but if $R_{0}\geq 1$, the disease will spread. In order to reduce $R_{0}$, we can vary the parameters in the expression for $R_{0}$. $R_{0}$ is dependent on these parameters; $\beta_{mf},\eta_{cm},\theta_{f},\eta_{sm},\beta_{fm}$, and $\eta_{cf},\theta_{m},\mu_{m},\eta_{sf},\gamma_{f},\alpha_{f},\mu_{f},\zeta_{f},\gamma_{m},\alpha_{m},\zeta_{m}$. If all the parameters are held constant, and we vary $\alpha_{f}$, we see that an increase in $\alpha_{f}$ will cause a decrease in $R_{0}$, and an increase in $\alpha_{m}$ will cause a decrease in $R_{0}$. The parameter, $\gamma_{f}$ which is the rate at which females move from the incubative class, $i_{cf}$, to the symptomatic class, $i_{sf}$ (progression rate in females) and $\gamma_{m}$ which is the rate at which males move from the incubative class, $i_{cm}$, to the symptomatic class, $i_{sm}$ (progression rate in males). Thus, if we increase $\gamma_{f}$, $\gamma_{m}$ will in turn decrease the value of $R_{0}$. Thus, there must be early detection of the contraction of the disease. We can also see that, the transmission rates $\beta_{mf},\beta_{fm},\eta_{cm},\eta_{sm},\eta_{cf}$, and $\eta_{cm}$ are directly proportional to the gonorrhea reproduction number. Hence, if the transmission rate $\beta_{mf}$ is decreased, $R_{0}$ will also decrease. Similarly, if we decrease $\beta_{fm}$, $R_{0}$ decreases. The impact of $\beta_{fm}$ and $\beta_{mf}$ is shown in Fig. 1 and Fig. 2, while the impact of $\alpha_{f}$ and $\alpha_{m}$ is shown in Fig. 3 and Fig. 4. Fig. 1a-Fig. 1d shows the impact of the female-to-male transmission on the gonorrhea reproduction number and its overall outcome on the infected compartments. Fig. 2a-Fig. 2d shows the impact of the male-to-female transmission on the gonorrhea reproduction number and its overall outcome on the infected compartments. Fig. 3a-Fig. 3d shows the impact of the male recovery on the gonorrhea reproduction number and its overall outcome on the infected compartments. Finally, Fig. 4a-Fig. 4d shows the impact of the female recovery on the gonorrhea reproduction number and its overall outcome on the infected compartments.

## 4 Sensitivity analysis

To study the optimal control model, we carried out a sensitivity analysis so to ascertain the parameters that contribute to the spread of the infection faster and the decline of the infection. Here, we will use the forward index sensitivity analysis (local sensitivity analysis) as defined in the following works [22], [23], [24], [25]. The forward sensitivity analysis is defined as

(6) $$\Theta_{h_{i}^{⁎}}^{R_{0}}=\frac{\partial R_{0}}{\partial h_{i}^{⁎}}\times \frac{h_{i}^{⁎}}{R_{0}},$$

where $h_{i}^{⁎}$ represents the various parameters in the gonorrhea reproduction number, $R_{0}$. Using the parameter values in Table 2, the forward (local) sensitivity indexes using equation (6) are represented in Table 1.

**Table 1:** Forward sensitivity analysis.

Parameters	Sensitivity index	Sign	Parameters	Sensitivity index	Sign
*θ*_*f*_	0.5000	+	*β*_*mf*_	0.5000	+
*θ*_*m*_	0.5000	+	*η*_*cf*_	0.1310	+
*η*_*sm*_	0.1854	+	*η*_*sf*_	0.3690	+
*μ*_*f*_	−0.7912	−	*η*_*cm*_	0.3146	+
*μ*_*m*_	−0.5835	−	*α*_*f*_	−0.1559	−
*α*_*m*_	−0.1682	−	*γ*_*f*_	−0.0477	−
*β*_*fm*_	0.5000	+	*γ*_*m*_	−0.2479	−
*ζ*_*f*_	−0.0052	−	*ζ*_*m*_	−4.2052 × 10^−4^	−

The analytic sensitivity expressions from equation (6) are given as follows:

$$\Theta_{\beta_{mf}}^{R_{0}}=\frac{1}{2},\;\;\Theta_{\theta_{f}}^{R_{0}}=\frac{1}{2},\;\;\Theta_{\beta_{fm}}^{R_{0}}=\frac{1}{2},\Theta_{\theta_{m}}^{R_{0}}=\frac{1}{2},\Theta_{\eta_{cm}}^{R_{0}}=\frac{\beta_{fm}\;\beta_{mf}\;\eta_{cm}\;\theta_{f}\;\theta_{m}\;\Delta_{1}}{\mu_{f}\;\mu_{m}\;\left(\gamma_{f}+\mu_{f}\right)\;\left(\gamma_{m}+\mu_{m}\right)\;\left(\alpha_{f}+\mu_{f}+\zeta_{f}\right)\Delta_{3}},\Theta_{\eta_{sm}}^{R_{0}}=\frac{\beta_{fm}\;\beta_{mf}\;\eta_{sm}\;\gamma_{m}\;\theta_{f}\;\theta_{m}\;\Delta_{1}}{\mu_{f}\;\mu_{m}\;\left(\gamma_{f}+\mu_{f}\right)\;\left(\gamma_{m}+\mu_{m}\right)\;\left(\alpha_{f}+\mu_{f}+\zeta_{f}\right)\;\left(\alpha_{m}+\mu_{m}+\zeta_{m}\right)\Delta_{3}},\Theta_{\eta_{cf}}^{R_{0}}=\frac{\beta_{fm}\;\beta_{mf}\;\eta_{cf}\;\theta_{f}\;\theta_{m}\;\Delta_{2}}{\mu_{f}\;\mu_{m}\;\left(\gamma_{f}+\mu_{f}\right)\;\left(\gamma_{m}+\mu_{m}\right)\;\left(\alpha_{m}+\mu_{m}+\zeta_{m}\right)\Delta_{4}},\Theta_{\eta_{sf}}^{R_{0}}=\frac{\beta_{fm}\;\beta_{mf}\;\eta_{sf}\;\gamma_{f}\;\theta_{f}\;\theta_{m}\;\Delta_{2}}{\mu_{f}\;\mu_{m}\;\left(\gamma_{f}+\mu_{f}\right)\;\left(\gamma_{m}+\mu_{m}\right)\;\left(\alpha_{f}+\mu_{f}+\zeta_{f}\right)\;\left(\alpha_{m}+\mu_{m}+\zeta_{m}\right)\Delta_{4}},\Theta_{\gamma_{f}}^{R_{0}}=-\frac{\gamma_{f}\;\left(\alpha_{f}\;\eta_{cf}+\eta_{cf}\;\mu_{f}-\eta_{sf}\;\mu_{f}+\eta_{cf}\;\zeta_{f}\right)}{2\;\left(\gamma_{f}+\mu_{f}\right)\;\left(\alpha_{f}\;\eta_{cf}+\eta_{sf}\;\gamma_{f}+\eta_{cf}\;\mu_{f}+\eta_{cf}\;\zeta_{f}\right)},\;\;\Theta_{\alpha_{f}}^{R_{0}}=-\frac{\alpha_{f}\;\eta_{sf}\;\gamma_{f}}{2\;\left(\alpha_{f}+\mu_{f}+\zeta_{f}\right)\;\left(\alpha_{f}\;\eta_{cf}+\eta_{sf}\;\gamma_{f}+\eta_{cf}\;\mu_{f}+\eta_{cf}\;\zeta_{f}\right)},\Theta_{\zeta_{f}}^{R_{0}}=-\frac{\eta_{sf}\;\gamma_{f}\;\zeta_{f}}{2\;\left(\alpha_{f}+\mu_{f}+\zeta_{f}\right)\;\left(\alpha_{f}\;\eta_{cf}+\eta_{sf}\;\gamma_{f}+\eta_{cf}\;\mu_{f}+\eta_{cf}\;\zeta_{f}\right)},\Theta_{\mu_{f}}^{R_{0}}=-\frac{\Delta_{8}}{2\;\left(\gamma_{f}+\mu_{f}\right)\;\left(\alpha_{f}+\mu_{f}+\zeta_{f}\right)\;\left(\alpha_{f}\;\eta_{cf}+\eta_{sf}\;\gamma_{f}+\eta_{cf}\;\mu_{f}+\eta_{cf}\;\zeta_{f}\right)},\Theta_{\mu_{m}}^{R_{0}}=-\frac{\Delta_{12}}{2\;\left(\gamma_{m}+\mu_{m}\right)\;\left(\alpha_{m}+\mu_{m}+\zeta_{m}\right)\;\left(\alpha_{m}\;\eta_{cm}+\eta_{sm}\;\gamma_{m}+\eta_{cm}\;\mu_{m}+\eta_{cm}\;\zeta_{m}\right)},\Theta_{\gamma_{m}}^{R_{0}}=-\frac{\gamma_{m}\;\left(\alpha_{m}\;\eta_{cm}+\eta_{cm}\;\mu_{m}-\eta_{sm}\;\mu_{m}+\eta_{cm}\;\zeta_{m}\right)}{2\;\left(\gamma_{m}+\mu_{m}\right)\;\left(\alpha_{m}\;\eta_{cm}+\eta_{sm}\;\gamma_{m}+\eta_{cm}\;\mu_{m}+\eta_{cm}\;\zeta_{m}\right)},\Theta_{\alpha_{m}}^{R_{0}}=-\frac{\alpha_{m}\;\eta_{sm}\;\gamma_{m}}{2\;\left(\alpha_{m}+\mu_{m}+\zeta_{m}\right)\;\left(\alpha_{m}\;\eta_{cm}+\eta_{sm}\;\gamma_{m}+\eta_{cm}\;\mu_{m}+\eta_{cm}\;\zeta_{m}\right)},\Theta_{\zeta_{m}}^{R_{0}}=-\frac{\eta_{sm}\;\gamma_{m}\;\zeta_{m}}{2\;\left(\alpha_{m}+\mu_{m}+\zeta_{m}\right)\;\left(\alpha_{m}\;\eta_{cm}+\eta_{sm}\;\gamma_{m}+\eta_{cm}\;\mu_{m}+\eta_{cm}\;\zeta_{m}\right)},$$

where,

$$\Delta_{1}=\alpha_{f}\;\eta_{cf}+\eta_{sf}\;\gamma_{f}+\eta_{cf}\;\mu_{f}+\eta_{cf}\;\zeta_{f},\Delta_{2}=\alpha_{m}\;\eta_{cm}+\eta_{sm}\;\gamma_{m}+\eta_{cm}\;\mu_{m}+\eta_{cm}\;\zeta_{m},\Delta_{3}=\;\frac{2\beta_{fm}\;\beta_{mf}\;\theta_{f}\;\theta_{m}\;\Delta_{1}\;\left(\alpha_{m}\;\eta_{cm}+\eta_{sm}\;\gamma_{m}+\eta_{cm}\;\mu_{m}+\eta_{cm}\;\zeta_{m}\right)}{\mu_{f}\;\mu_{m}\;\left(\gamma_{f}+\mu_{f}\right)\;\left(\gamma_{m}+\mu_{m}\right)\;\left(\alpha_{f}+\mu_{f}+\zeta_{f}\right)\;\left(\alpha_{m}+\mu_{m}+\zeta_{m}\right)},\Delta_{4}=\;\frac{2\beta_{fm}\;\beta_{mf}\;\theta_{f}\;\theta_{m}\;\left(\alpha_{f}\;\eta_{cf}+\eta_{sf}\;\gamma_{f}+\eta_{cf}\;\mu_{f}+\eta_{cf}\;\zeta_{f}\right)\;\Delta_{2}}{\mu_{f}\;\mu_{m}\;\left(\gamma_{f}+\mu_{f}\right)\;\left(\gamma_{m}+\mu_{m}\right)\;\left(\alpha_{f}+\mu_{f}+\zeta_{f}\right)\;\left(\alpha_{m}+\mu_{m}+\zeta_{m}\right)},\Delta_{5}=2\;\eta_{cf}\;\mu_{f}^{3}+\eta_{cf}\;\gamma_{f}\;\zeta_{f}^{2}+\eta_{sf}\;\gamma_{f}^{2}\;\zeta_{f}+2\;\eta_{cf}\;\mu_{f}\;\zeta_{f}^{2},\Delta_{6}=4\;\eta_{cf}\;\mu_{f}^{2}\;\zeta_{f}+\alpha_{f}^{2}\;\eta_{cf}\;\gamma_{f}+\alpha_{f}\;\eta_{sf}\;\gamma_{f}^{2}+4\;\alpha_{f}\;\eta_{cf}\;\mu_{f}^{2}+2\;\alpha_{f}^{2}\;\eta_{cf}\;\mu_{f}+\eta_{cf}\;\gamma_{f}\;\mu_{f}^{2}+3\;\eta_{sf}\;\gamma_{f}\;\mu_{f}^{2},\Delta_{7}=4\;\alpha_{f}\;\eta_{cf}\;\mu_{f}\;\zeta_{f}+2\;\eta_{cf}\;\gamma_{f}\;\mu_{f}\;\zeta_{f}+2\;\eta_{sf}\;\gamma_{f}\;\mu_{f}\;\zeta_{f}+2\;\alpha_{f}\;\eta_{cf}\;\gamma_{f}\;\mu_{f}+2\;\alpha_{f}\;\eta_{sf}\;\gamma_{f}\;\mu_{f},\Delta_{8}=\Delta_{5}+\Delta_{6}+\Delta_{7}+2\;\eta_{sf}\;\gamma_{f}^{2}\;\mu_{f}+2\;\alpha_{f}\;\eta_{cf}\;\gamma_{f}\;\zeta_{f},\Delta_{9}=2\;\eta_{cm}\;\mu_{m}^{3}+\eta_{cm}\;\gamma_{m}\;\zeta_{m}^{2}+\eta_{sm}\;\gamma_{m}^{2}\;\zeta_{m}+2\;\eta_{cm}\;\mu_{m}\;\zeta_{m}^{2},\Delta_{10}=4\;\eta_{cm}\;\mu_{m}^{2}\;\zeta_{m}+\alpha_{m}^{2}\;\eta_{cm}\;\gamma_{m}+\alpha_{m}\;\eta_{sm}\;\gamma_{m}^{2}+4\;\alpha_{m}\;\eta_{cm}\;\mu_{m}^{2}+2\;\alpha_{m}^{2}\;\eta_{cm}\;\mu_{m}+\eta_{cm}\;\gamma_{m}\;\mu_{m}^{2}+3\;\eta_{sm}\;\gamma_{m}\;\mu_{m}^{2},\Delta_{11}=+2\;\eta_{sm}\;\gamma_{m}^{2}\;\mu_{m}+2\;\alpha_{m}\;\eta_{cm}\;\gamma_{m}\;\zeta_{m}+4\;\alpha_{m}\;\eta_{cm}\;\mu_{m}\;\zeta_{m}+2\;\eta_{cm}\;\gamma_{m}\;\mu_{m}\;\zeta_{m}+2\;\eta_{sm}\;\gamma_{m}\;\mu_{m}\;\zeta_{m},\Delta_{12}=\Delta_{9}+\Delta_{10}+\Delta_{11}+2\;\alpha_{m}\;\eta_{cm}\;\gamma_{m}\;\mu_{m}+2\;\alpha_{m}\;\eta_{sm}\;\gamma_{m}\;\mu_{m}.$$

## 5 Optimal control formulation

Following the sensitivity analysis in Table 1, this part applies optimal control theory to the gonorrhoea model. As shown in Table 1, the transmission and relative infectivity rates promote the spread of infection. In contrast, recovery rates reduce disease transmission, which is also essential to consider when managing disease spread. Therefore, we consider four crucial controls to reduce gonorrhea infection in any community.

The controls to be implemented are;

1. $u_{1}$ = Educating people about gonorrhea and its transmission.
2. $u_{2}$ = Condom usage during sexual intercourse.
3. $u_{3}$ = Vaccination against the contraction of gonorrhea.
4. $u_{4}$ = Treatment in both populations.

Now, let the model system be $y^{\prime }(t)$ = f(y(t)) with initial conditions, y(0) = $y_{0}$ $\in R_{+}^{8}$, where $y:[0,\infty ]\mapsto R_{+}^{8}$. We have u(t) = $(u_{1},u_{2},u_{3},u_{4})\in U$ representing the controls to be implemented on the model and the admissible control set given by *U* = $\left\{(u_{1},u_{2},u_{3},u_{4})|u_{i}\;\text{is Lebesgue measurable on}\;[0,1],0\leq u_{i}(t)\leq 1,t_{0}\leq t\leq t_{f}\right\}$. We aim to find the best control to minimize the number of females and males in the respective incubative classes, the number of females and males in the respective symptomatic classes and the number of males and females in the respective recovery classes. The objective functional we want to minimize is given by

(7) $$J(u_{1},u_{2},u_{3},u_{4})=\text{min}\int_{t_{0}}^{t_{f}}\left[i_{c}f+i_{sf}+i_{cm}+i_{sm}+r_{f}+r_{m}+\frac{G_{1}}{2}u_{1}^{2}+\frac{G_{2}}{2}u_{2}^{2}+\frac{G_{3}}{2}u_{3}^{2}+\frac{G_{4}}{2}u_{4}^{2}\right]dt,$$

and it is subject to the constraints,

(8) $$\left\{ \begin{array}{cc} \frac{ds_{f}}{dt} & =\theta_{f}-(1-(u_{1}+u_{2}+u_{3}))\beta_{mf}(\eta_{sm}i_{sm}+\eta_{cm}i_{cm})s_{f}+\rho_{f}r_{f}-\mu_{f}s_{f},\\ \frac{di_{c}f}{dt} & =(1-(u_{1}+u_{2}+u_{3}))\beta_{mf}(\eta_{sm}i_{sm}+\eta_{cm}i_{cm})s_{f}-(\gamma_{f}+\mu_{f})i_{c}f+(1-\rho_{f})\kappa_{f}r_{f},\\ \frac{di_{sf}}{dt} & =\gamma_{f}i_{c}f-(u_{4}+\mu_{f}+\zeta_{f})i_{sf},\\ \frac{dr_{f}}{dt} & =u_{4}i_{sf}-(\rho_{f}+(1-\rho_{f})\kappa_{f}+\mu_{f})r_{f},\\ \frac{ds_{m}}{dt} & =\theta_{m}-(1-(u_{1}+u_{2}+u_{3}))\beta_{fm}(\eta_{sf}i_{sf}+\eta_{c}fi_{c}f)s_{m}+\rho_{m}r_{m}-\mu_{m}s_{m},\\ \frac{di_{cm}}{dt} & =(1-(u_{1}+u_{2}+u_{3}))\beta_{fm}(\eta_{sf}i_{sf}+\eta_{c}fi_{c}f)s_{m}-(\gamma_{m}+\mu_{m})i_{cm}+\rho_{m}\kappa_{m}r_{m},\\ \frac{di_{sm}}{dt} & =\gamma_{m}i_{c}f-(u_{4}+\mu_{m}+\zeta_{m})i_{sm},\\ \frac{dr_{m}}{dt} & =u_{4}i_{sm}-(\rho_{m}+(1-\rho_{m})\kappa_{m}+\mu_{m})r_{m}, \end{array}\right.$$

where, $s_{f}(t)\geq 0,\;i_{c}f(t)\geq 0,\;i_{sf}(t)\geq 0,\;i_{f}(t)\geq 0,\;i_{m}(t)\geq 0,\;i_{cm}(t)\geq 0,\;i_{sm}(t)\geq 0,\;r_{m}(t)\geq 0$.

From equation (7) and equation (8), we assume that the weight constants associated with the state variables are one. The term $G_{i},i=1,2,3,4$, represents the relative importance of the respective controls on the model.

The half is the factor that reduces the effect of the controls, and the squared denotes the non-linearity of the controls.

We need to show that the optimal control problem exists. To do this, we use the Fillippove-Cesari theorem as used by [26]. The optimal control problem exists if;

1. The admissible control set, *U* is bounded and closed.
2. The state variables and the control set are non-empty.
3. The state system differential equations are bounded by a linear function in the state and control variables.
4. The integrand of the objective functional is convex.

It can be seen by the definition of the admissible control set *U* that it is bounded and closed.

To show that the integrand of the objective functional is convex, we find the Hessian matrix with respect to *u*, which is a matrix of second-order partial derivatives.

$$\left[\begin{array}{cccc} G_{1} & 0 & 0 & 0\\ 0 & G_{2} & 0 & 0\\ 0 & 0 & G_{3} & 0\\ 0 & 0 & 0 & G_{4} \end{array}\right]$$

Since all the diagonals and the leading principal minors of the Hessian matrix are positive, it implies it is strictly convex and hence the objective functional is strictly convex. Using Pontryagin's maximum principle, we find the Hamiltonian function with state variables, $i_{cf}$ = $i_{cf}^{⁎}$, $i_{sf}$ = $i_{sf}^{⁎}$, $i_{cm}$ = $i_{cm}^{⁎}$, $i_{sm}$ = $i_{sm}^{⁎}$, $r_{f}$ = $r_{f}^{⁎}$, $r_{m}$ = $r_{m}^{⁎}$.

$$H=\;i_{c}^{⁎}f+i_{sf}^{⁎}+i_{cm}^{⁎}+i_{sm}^{⁎}+r_{f}^{⁎}+r_{m}^{⁎}+\frac{G_{1}}{2}u_{1}^{2}+\frac{G_{2}}{2}u_{2}^{2}+\frac{G_{3}}{2}u_{3}^{2}+\frac{G_{4}}{2}u_{4}^{2}+\;\lambda_{1}[\theta_{f}-(1-(u_{1}+u_{2}+u_{3}))\beta_{mf}(\eta_{sm}i_{sm}^{⁎}+\eta_{cm}i_{cm}^{⁎})s_{f}^{⁎}+\rho_{f}r_{f}^{⁎}-\mu_{f}s_{f}^{⁎}]+\;\lambda_{2}[(1-(u_{1}+u_{2}+u_{3}))\beta_{mf}(\eta_{sm}i_{sm}^{⁎}+\eta_{cm}i_{cm}^{⁎})s_{f}^{⁎}-(\gamma_{f}+\mu_{f})i_{cf}^{⁎}+(1-\rho_{f})\kappa_{f}r_{f}^{⁎}]+\;\lambda_{3}[\gamma_{f}i_{cf}^{⁎}-(u_{4}+\mu_{f}+\zeta_{f})i_{sf}^{⁎}]+\;\lambda_{4}[u_{4}i_{sf}^{⁎}-(\rho_{f}+(1-\rho_{f})\kappa_{f}+\mu_{f})r_{f}^{⁎}]+\;\lambda_{5}[\theta_{f}-(1-(u_{1}+u_{2}+u_{3}))\beta_{fm}(\eta_{sf}i_{sf}^{⁎}+\eta_{cf}i_{cf}^{⁎})s_{m}^{⁎}+\rho_{m}r_{m}^{⁎}-\mu_{m}s_{m}^{⁎}]+\;\lambda_{6}[(1-(u_{1}+u_{2}+u_{3}))\beta_{fm}(\eta_{sf}i_{sf}^{⁎}+\eta_{cf}i_{cf}^{⁎})s_{m}^{⁎}-(\gamma_{m}+\mu_{m})i_{cm}^{⁎}+(1-\rho_{m})\kappa_{m}r_{m}^{⁎}]+\;\lambda_{7}[\gamma_{m}i_{cm}^{⁎}-(u_{4}+\mu_{m}+\zeta_{m})i_{sm}^{⁎}]+\;\lambda_{8}[u_{4}i_{sm}^{⁎}-(\rho_{m}+(1-\rho_{m})\kappa_{m}+\mu_{m})r_{m}^{⁎}].$$

We consider the existence of the adjoint function $\lambda_{i}$ = 0, *i* = 1,2,…,8.

$$\begin{array}{cc} \frac{d\lambda_{1}}{dt}= & -\frac{\partial H}{\partial s_{f}^{⁎}}\\ = & \lambda_{1}[(1-(u_{1}+u_{2}+u_{3}))\beta_{mf}(\eta_{sm}i_{sm}+\eta_{cm}i_{cm})-\mu_{f}]-\lambda_{2}[(1-(u_{1}+u_{2}+u_{3}))\beta_{mf}(\eta_{sm}i_{sm}+\eta_{cm}i_{cm})],\\ \frac{d\lambda_{2}}{dt}= & -\frac{\partial H}{\partial i_{c}^{⁎}f}\\ = & -1+\lambda_{2}(\gamma_{f}+\mu_{f})-\lambda_{3}\gamma_{f}+\lambda_{5}[(1-(u_{1}+u_{2}+u_{3}))\beta_{fm}\eta_{cf}s_{m}^{⁎}]-\lambda_{6}[(1-(u_{1}+u_{2}+u_{3}))\beta_{fm}\eta_{cf}s_{m}^{⁎}],\\ \frac{d\lambda_{3}}{dt}= & -\frac{\partial H}{\partial i_{sf}^{⁎}}\\ = & -1+\lambda_{3}(u_{4}+\mu_{f}+\zeta_{f})-\lambda_{4}u_{4}+\lambda_{5}[(1-(u_{1}+u_{2}+u_{3}))\beta_{fm}\eta_{sm}s_{m}^{⁎}]-\lambda_{6}[(1-(u_{1}+u_{2}+u_{3}))\beta_{fm}\eta_{sm}s_{m}^{⁎}],\\ \frac{d\lambda_{4}}{dt}= & -\frac{\partial H}{\partial r_{f}^{⁎}},\\ = & -1-\lambda_{1}\rho_{f}-\lambda_{2}\rho_{f}+\lambda_{4}(\rho_{f}+(1-\rho_{f})\kappa_{f}+\mu_{f}),\\ \frac{d\lambda_{5}}{dt}= & -\frac{\partial H}{\partial s_{m}^{⁎}},\\ = & \lambda_{5}[(1-(u_{1}+u_{2}+u_{3}))\beta_{fm}(\eta_{sf}i_{sf}^{⁎}+\eta_{cf}i_{cf}^{⁎})+\mu_{m}]-\lambda_{6}[(1-(u_{1}+u_{2}+u_{3}))\beta_{fm}(\eta_{sf}i_{sf}^{⁎}+\eta_{cf}i_{cf}^{⁎})],\\ \frac{d\lambda_{6}}{dt}= & -\frac{\partial H}{\partial i_{cm}^{⁎}}\\ = & -1+\lambda_{1}[(1-(u_{1}+u_{2}+u_{3}))\beta_{mf}\eta_{cm}s_{f}^{⁎}]-\lambda_{2}[(1-(u_{1}+u_{2}+u_{3}))\beta_{mf}\eta_{cm}s_{f}^{⁎}]+\lambda_{6}(\gamma_{m}+\mu_{m})-\lambda_{7}\gamma_{m},\\ \frac{d\lambda_{7}}{dt}= & -\frac{\partial H}{\partial i_{sm}^{⁎}}\\ = & -1+\lambda_{1}[(1-(u_{1}+u_{2}+u_{3}))\beta_{mf}\eta_{sf}s_{f}^{⁎}]-\lambda_{2}[(1-(u_{1}+u_{2}+u_{3}))\beta_{mf}\eta_{sf}s_{f}^{⁎}]+\lambda_{7}(u_{4}+\mu_{m}+\zeta_{m})-\lambda_{8}u_{4},\\ \frac{a\lambda_{8}}{dt}= & -\frac{\partial H}{\partial r_{m}^{⁎}}\\ = & -\lambda_{5}\rho_{m}-\lambda_{6}\rho_{m}+\lambda_{8}(\rho_{m}+(1-\rho_{m})\kappa_{m}+\mu_{m}), \end{array}$$

with transversality condition $\lambda_{i}(t_{f})$ = 0, *i* = 1,2,…,8, $\forall u_{i}$,

$$\frac{\partial H}{\partial u_{i}}=0,i=1,2,3,4.$$

$${\frac{\partial H}{\partial u_{1}}|}_{u_{1}=u_{1}^{⁎}}=G_{1}u_{1}^{⁎}+\lambda_{1}\beta_{mf}(\eta_{sm}i_{sm}^{⁎}+\eta_{cm}i_{cm}^{⁎})s_{f}^{⁎}-\lambda_{2}\beta_{mf}(\eta_{sm}i_{sm}^{⁎}+\eta_{cm}i_{cm}^{⁎})s_{f}^{⁎}+\lambda_{5}\beta_{fm}(\eta_{sf}i_{sf}^{⁎}+\eta_{cf}i_{cf}^{⁎})s_{m}^{⁎}-\lambda_{6}\beta_{fm}(\eta_{sf}i_{sf}^{⁎}+\eta_{cf}i_{cf}^{⁎})s_{m}^{⁎}=0,u_{1}^{⁎}=\frac{(\lambda_{2}-\lambda_{1})\beta_{mf}(\eta_{sm}i_{sm}^{⁎}+\eta_{cm}i_{cm}^{⁎})s_{f}^{⁎}+(\lambda_{6}-\lambda_{5})\beta_{fm}(\eta_{sf}i_{sf}^{⁎}+\eta_{cf}i_{cf}^{⁎})s_{m}^{⁎}}{G_{1}}.{\frac{\partial H}{\partial u_{2}}|}_{u_{2}=u_{2}^{⁎}}=G_{2}u_{2}^{⁎}+\lambda_{1}\beta_{mf}(\eta_{sm}i_{sm}^{⁎}+\eta_{cm}i_{cm}^{⁎})s_{f}^{⁎}-\lambda_{2}\beta_{mf}(\eta -smi_{sm}^{⁎}+\eta_{cm}i_{cm}^{⁎})s_{f}^{⁎}+\lambda_{5}\beta_{fm}(\eta_{sf}i_{sf}^{⁎}+\eta_{cf}i_{cf}^{⁎})s_{m}^{⁎}-\lambda_{6}\beta_{fm}(\eta_{sf}i_{sf}^{⁎}+\eta_{cf}i_{cf}^{⁎})s_{m}^{⁎}=0,u_{2}^{⁎}=\frac{(\lambda_{2}-\lambda_{1})\beta_{mf}(\eta_{sm}i_{sm}^{⁎}+\eta_{cm}i_{cm}^{⁎})s_{f}^{⁎}+(\lambda_{6}-\lambda_{5})\beta_{fm}(\eta_{sf}i_{sf}^{⁎}+\eta_{cf}i_{cf}^{⁎})s_{m}^{⁎}}{G_{2}}.{\frac{\partial H}{\partial u_{3}}|}_{u_{3}=u_{3}^{⁎}}=G_{3}u_{3}^{⁎}+\lambda_{1}\beta_{mf}(\eta_{sm}i_{sm}^{⁎}+\eta_{cm}i_{cm}^{⁎})s_{f}^{⁎}-\lambda_{2}\beta_{mf}(\eta_{sm}i_{sm}^{⁎}+\eta_{cm}i_{cm}^{⁎})s_{f}^{⁎}+\lambda_{5}\beta_{fm}(\eta_{sf}i^{⁎}sf+\eta_{cf}i_{cf}^{⁎})s_{m}^{⁎}-\lambda_{6}\beta_{fm}(\eta_{sf}i_{sf}^{⁎}+\eta_{cf}i_{cf}^{⁎})s_{m}^{⁎}=0,u_{3}^{⁎}=\frac{(\lambda_{2}-\lambda_{1})\beta_{mf}(\eta_{sm}i_{sm}^{⁎}+\eta_{cm}i_{cm}^{⁎})s_{f}^{⁎}+(\lambda_{6}-\lambda_{5})\beta_{fm}(\eta_{sf}i_{sf}^{⁎}+\eta_{cf}i_{cf}^{⁎})s_{m}^{⁎}}{G_{3}}.{\frac{\partial H}{\partial u_{4}}|}_{u_{4}=u_{4}^{⁎}}=G_{4}u_{4}^{⁎}-\lambda_{3}i_{sf}^{⁎}+\lambda_{4}i_{sf}^{⁎}-\lambda_{7}i_{sm}^{⁎}+\lambda_{8}i_{sm}^{⁎}=0,u_{4}^{⁎}=\frac{\lambda_{3}i_{sf}^{⁎}-\lambda_{4}i_{sf}^{⁎}+\lambda_{7}i_{sm}^{⁎}-\lambda_{8}i_{sm}^{⁎}}{G_{4}}.$$

Then the optimal control strategies taking into account the variation arguments are;

$$u_{1}^{⁎}=min\left\{max\left(0,\frac{(\lambda_{2}-\lambda_{1})\beta_{mf}(\eta_{sm}i_{sm}^{⁎}+\eta_{cm}i_{cm}^{⁎})s_{f}^{⁎}+(\lambda_{6}-\lambda_{5})\beta_{fm}(\eta_{sf}i_{sf}^{⁎}+\eta_{cf}i_{cf}^{⁎})s_{m}^{⁎}}{G_{1}}\right),u_{1max}\right\},u_{2}^{⁎}=min\left\{max\left(0,\frac{(\lambda_{2}-\lambda_{1})\beta_{mf}(\eta_{sm}i_{sm}^{⁎}+\eta_{cm}i_{cm}^{⁎})s_{f}^{⁎}+(\lambda_{6}-\lambda_{5})\beta_{fm}(\eta_{sf}i_{sf}^{⁎}+\eta_{cf}i_{cf}^{⁎})s_{m}^{⁎}}{G_{2}}\right),u_{2max}\right\},u_{3}^{⁎}=min\left\{max\left(0,\frac{(\lambda_{2}-\lambda_{1})\beta_{mf}(\eta_{sm}i_{sm}^{⁎}+\eta_{cm}i_{cm}^{⁎})s_{f}^{⁎}+(\lambda_{6}-\lambda_{5})\beta_{fm}(\eta_{sf}i_{sf}^{⁎}+\eta_{cf}i_{cf}^{⁎})s_{m}^{⁎}}{G_{3}}\right),u_{3max}\right\},u_{4}^{⁎}=min\left\{max\left(0,\frac{\lambda_{3}i_{sf}^{⁎}-\lambda_{4}i_{sf}^{⁎}+\lambda_{7}i_{sm}^{⁎}-\lambda_{8}i_{sm}^{⁎}}{G_{4}}\right),u_{4max}\right\}.$$

## 6 Numerical simulations

**Figure 5:**
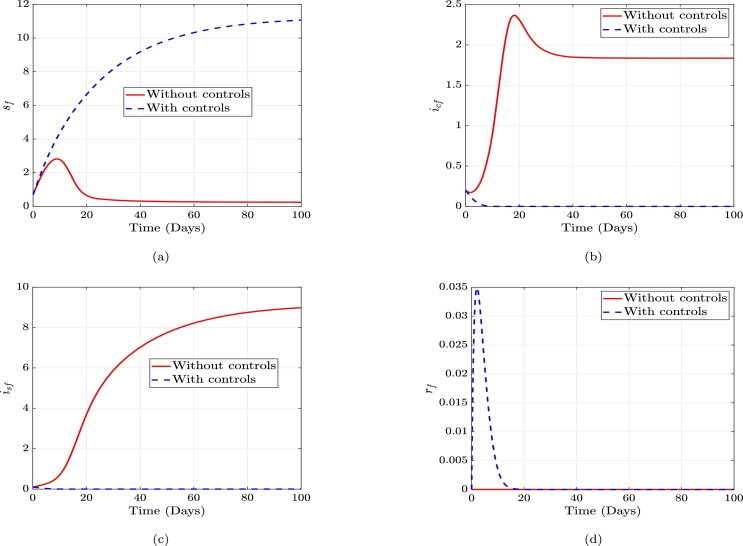
Without controls and with controls plots of the susceptible, incubative, symptomatic and recovered females, respectively.

**Figure 6:**
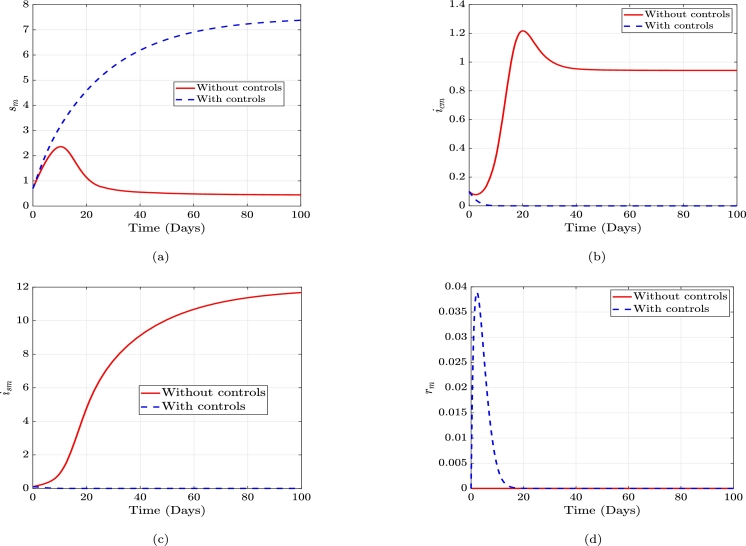
Without controls and with controls plots of the susceptible, incubative, symptomatic and recovered males, respectively.

We perform numerical simulations to visualize the effects of implementing the controls discussed earlier. We use the Range-Kutta fourth-order method and the parameter values in Table 2. Using the Forward-Backward sweep method proposed by [27] with the aid of Matlab (2021a). We used a period of 100 days with the following initial values for the state variables, $s_{f}(0)=0.7$, $i_{c}f(0)=0.2$, $i_{sf}(0)=0.1$, $r_{f}(0)=0$, $s_{m}(0)=0.8$, $i_{cm}(0)=0.1$, $i_{sm}=0.1$ and $r_{m}(0)=0$. The weight constants associated with the controls, $u_{1},u_{2},u_{3},u_{4}$, are considered to be equal thus $G_{1}=G_{2}=G_{3}=G_{4}=1$. In Fig. 5 and Fig. 6, the red line represents the population without the stated controls and the dashed blue line represents the population with the stated controls.

**Table 2:** Parameter Table.

Parameters	Values, day^−1^	Reference	Parameters	Values, day^−1^	Reference
*θ*_*f*_	0.45	[12]	*β*_*mf*_	0.15	[28]
*θ*_*m*_	0.3	[12]	*η*_*c*_*f*	0.65	Assumed
*η*_*sm*_	0.4	Assumed	*η*_*sf*_	0.65	Assumed
*μ*_*f*_	0.04	[11]	*η*_*cm*_	0.4	Assumed
*μ*_*m*_	0.04	[11]	*ρ*_*f*_	0.04	[11]
*α*_*f*_	0.03	[28]	*ρ*_*m*_	0.04	[11]
*κ*_*f*_	0.01	Assumed	*κ*_*m*_	0.01	Assumed
*α*_*m*_	0.4	[28]	*γ*_*f*_	0.2	Assumed
*β*_*fm*_	0.0625	[28]	*γ*_*m*_	0.26	Assumed
*ζ*_*f*_	0.001	Assumed	*ζ*_*m*_	0.001	Assumed

## 7 Result and discussion

The optimal control model of gonorrhea has been numerically solved and the results have been displayed graphically.

In Fig. 5, the susceptible female class without controls decreases as more individuals will contract the disease; however, when these four controls are implemented, the female susceptible class increases. Thus, the number of healthy individuals increases because most people either protect themselves from contracting the disease, and those who have are also treated. In Figure (5b), the female incubative population grows when there are no controls. After implementing the controls, the number of persons in the female incubative class reduces. Considering the female symptomatic class in Figure (5c), when the controls are introduced, it curbs the spread of the disease; thus, few people contract the disease, but without these controls, the number of individuals in symptomatic class grows which indicates more transmissions in the absence of the controls. The recovered female class in Figure (5d) without controls is zero, meaning no one will recover from this disease without these controls. However, after the implementation of these controls, the number of recovered individuals increases. It can be seen that within a short period after recovery, the graph begins to decline. This is a result of the nature of the disease. Thus, there is no permanent immunity; hence after recovery, the individuals in this class become susceptible again.

The graphs in Fig. 6 represent the dynamics in the male population with and without controls. It is similar to Fig. 5. Thus, in the male susceptible class in Figure (6a), the individuals in this class will decrease if there are no controls. Upon introducing the controls in this class, the susceptible males increase, which indicates more healthy males and less contraction of the disease. The male incubative class in Figure (6b) with controls decreases, indicating less contraction of the disease and increases when the controls are implemented. In the symptomatic male class in Figure (6c), there is an increase when there are no controls and a decrease after introducing the control. The recovered male class in Figure (6d) without controls is zero as in the female population, and it increases when the controls are implemented. Since recovery is temporal (no permanent immunity), the individuals in this class become susceptible again after recovery.

**Figure 7:**
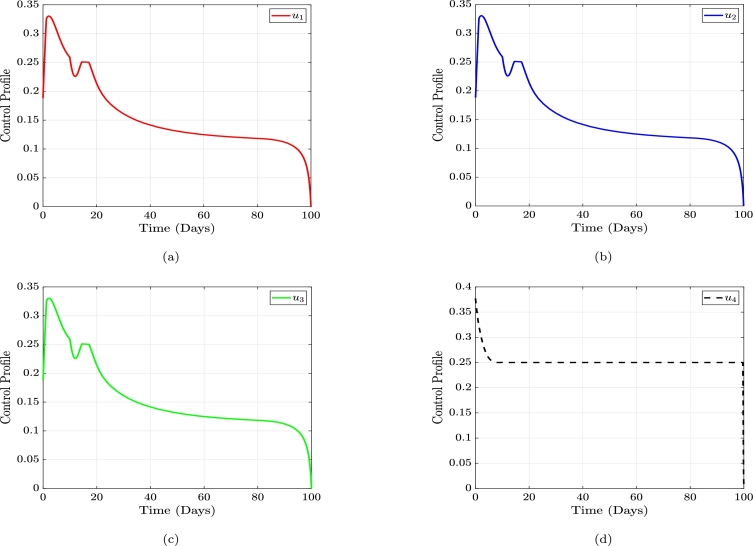
The dynamical trajectory of the control profile plot of education (*u*_1_), condom usage (*u*_2_), vaccination (*u*_3_) and treatment (*u*_4_), respectively.

Fig. 7, represents the graphs of the optimal control profile, which shows how to implement each control in order to attain the expected results. From Figure, (7a), the control $u_{1}$ should start from around 0.2 and it should increase gradually to around 0.33 within the first 3 days. It should then be decreased to around 0.22 by day 11 and finally increased gradually to 0.25 by day 14. This should be maintained for about 2 days, after which it should be gradually decreased until around day 94. After this day, it should not be decreased further. The graphs, Figure (7b) and Figure (7c) follow a similar trajectory which indicates that the controls $u_{2}$, and $u_{3}$ should all follow the same procedure as the control $u_{1}$. In Figure (7d), we have that the control $u_{4}$ should start at around 0.37 on the first day. This should be gradually decreased to 0.25 by day 9, and afterwards, it should be maintained until day 99, after which it should be maintained.

**Figure 8:**
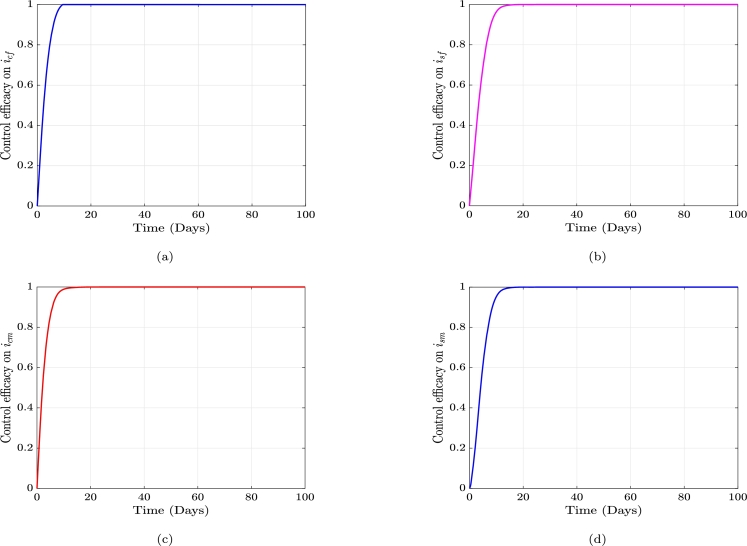
The dynamical trajectory of the control efficacy plot of infected females and males compartments, respectively.

The graphs in Fig. 8 gives us the efficacy plots, which depict how efficient these controls are. From the respective, Fig. 8a, Fig. 8b, Fig. 8c and Fig. 8d, it can be seen that the controls implemented achieved a 100% efficacy on the disease classes (i.e. $i_{c}f$, $i_{sf}$, $i_{cm}$, $i_{sm}$) after the first 11 days.

## 8 Conclusion

Using a heterogeneous population model, we transformed the model developed by [5] in this paper. We calculated the gonorrhoea reproduction number $R_{0}$ and the gonorrhea-free equilibrium point of the model. The gonorrhoea model's optimality system, which demonstrates the circumstances required to enhance gonorrhoea control, is constructed using Pontryagin's Maximum Principle. The controls that were considered included educating people about gonorrhea and how it spread $u_{1}$, use of condoms during sexual activity $u_{2}$, vaccination $u_{3}$ and treatment $u_{4}$ for both populations. When available, we used existing literature to source the parameters for our optimum control analysis; otherwise, we made assumptions to match the model analysis for illustration purposes. Furthermore, as indicated by the numerical solution results, the optimal method was effective when all the controls $u_{1}$, $u_{2}$, $u_{3}$, and $u_{4}$ were implemented on both the male and female compartments. The efficacy plots provided by the figures in Fig. 8 show the effectiveness of these controls. According to the graphs, after the first 11 days, the controls had a 100% efficacy rate when implemented in the disease classes (i.e. $i_{cf}$, $i_{sf}$, $i_{cm}$, $i_{sm}$). For further research, we intend to investigate the fractional and fractal analysis of this gonorrhea model.

## CRediT authorship contribution statement

Joshua Kiddy K. Asamoah, Beilawu Safianu, Emmanuel Afrifa, Benjamin Obeng, Baba Seidu, Fredrick Asenso Wireko, and Gui-Quan Sun: Conceived and designed the experiment; Performed the experiment; Analysis and interpreted the data; materials; analysis tools or data; Wrote the paper.

## Declaration of Competing Interest

The authors declare that they have no known competing financial interests or personal relationships that could have appeared to influence the work reported in this paper.

## Data Availability

No data was used for the research described in the article.
